# Autosomal recessive bestrophinopathy associated with angle-closure glaucoma

**DOI:** 10.1007/s10633-014-9444-z

**Published:** 2014-05-24

**Authors:** C. Crowley, R. Paterson, T. Lamey, T. McLaren, J. De Roach, E. Chelva, J. Khan

**Affiliations:** 1 Department of Medical Technology and Physics, Sir Charles Gairdner Hospital, Hospital Avenue, Nedlands, WA 6009 Australia; 2 Centre for Ophthalmology and Visual Science, University of Western Australia, Nedlands, WA 6009 Australia

**Keywords:** *BEST1* gene, Autosomal recessive bestrophinopathy, Best vitelliform macular dystrophy, Angle-closure glaucoma, Electroretinography, Electro-oculography, Intraocular pressure

## Abstract

**Purpose:**

Abnormalities in the *BEST1* gene have recently been recognised as causing autosomal recessive bestrophinopathy (ARB). ARB has been noted to have a variable phenotypic presentation, distinct from that of autosomal dominant Best vitelliform macular dystrophy (BVMD). Both conditions are associated with deposits in the retina, a reduced or absent electro-oculography (EOG) light rise, and the risk of developing angle-closure glaucoma. Herein, we describe the clinical and genetic characteristics of a young male diagnosed with ARB associated with angle-closure glaucoma resulting from a novel homozygous mutation in *BEST1*.

**Methods:**

All research involved in this case adhered to the tenets of the Declaration of Helsinki. The proband underwent slitlamp examination, retinal autofluorescence imaging and optical coherence tomography after presenting with deteriorating vision. The findings prompted genetic testing with bi-directional DNA sequencing of coding and flanking intronic regions of *BEST1*. The proband’s family members were subsequently screened.

**Results:**

A provisional diagnosis of ARB was made based on the findings of subretinal and schitic lesions on fundoscopy and retinal imaging, together with abnormal EOG and electroretinography. Genetic testing identified a novel homozygous mutation in *BEST1*, c.636+1 G>A. Family members were found to carry one copy of the mutation and had no clinical or electrophysiological evidence of disease. The proband was additionally diagnosed with angle-closure glaucoma requiring topical therapy, peripheral iridotomies and phacoemulsification.

**Conclusions:**

Phenotypic overlap, reduced penetrance, variable expressivity and the ongoing discovery of new forms of bestrophinopathies add to the difficulty in distinguishing these retinal diseases. All patients diagnosed with ARB or BVMD should be examined for narrow angles and glaucoma, given their frequent association with these conditions.

## Introduction

Autosomal recessive bestrophinopathy (ARB) has recently been described and has a more global influence on eye development and physiology than autosomal dominant Best vitelliform macular dystrophy (BVMD), otherwise known as Best disease. We present a case of ARB associated with narrow-angle glaucoma and a novel homozygous mutation in *BEST1*.

## Case description

**Fig. 1:**
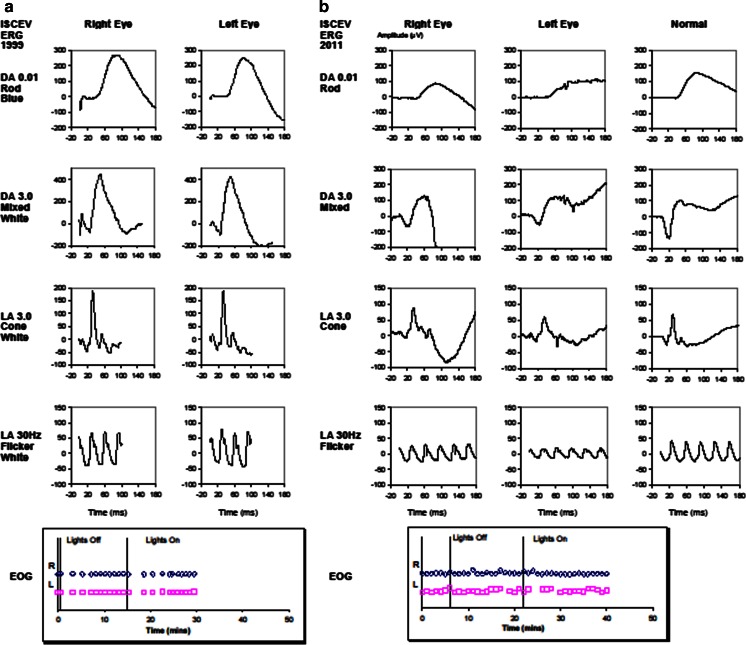
**a** 1999 electrophysiology which showed normal dark-adapted ERG but abnormal flicker ERG with b-wave delay and grossly abnormal EOG. Recordings were made on a custom built electrophysiology system according to current ISCEV standards at the time. The ERG was measured with Burian-Allen corneal contact lens electrodes. **b** 2011 electrophysiology which showed deterioration in the dark-adapted ERG with delayed and reduced responses, whilst light-adapted cone and flicker ERG were further delayed. The EOG remained grossly abnormal. Recordings were made on the LKC EMWIN E-3000 System according to current ISCEV standards. The ERG was measured with HK-Loop electrodes

A 26-year-old male presented with deteriorating vision in both eyes. He had no family history of eye disorders but had been diagnosed with possible Stargardt disease at the age of 12 years. This diagnosis was amended to exudative polymorphous vitelliform macular dystrophy two years later when he underwent electrophysiological studies which revealed normal rod electroretinography (ERG) but abnormal flicker ERG with b-wave delay and abnormal electro-oculography (EOG) (Fig. 1a). A trial of steroids had no benefit at that time.

**Fig. 2:**
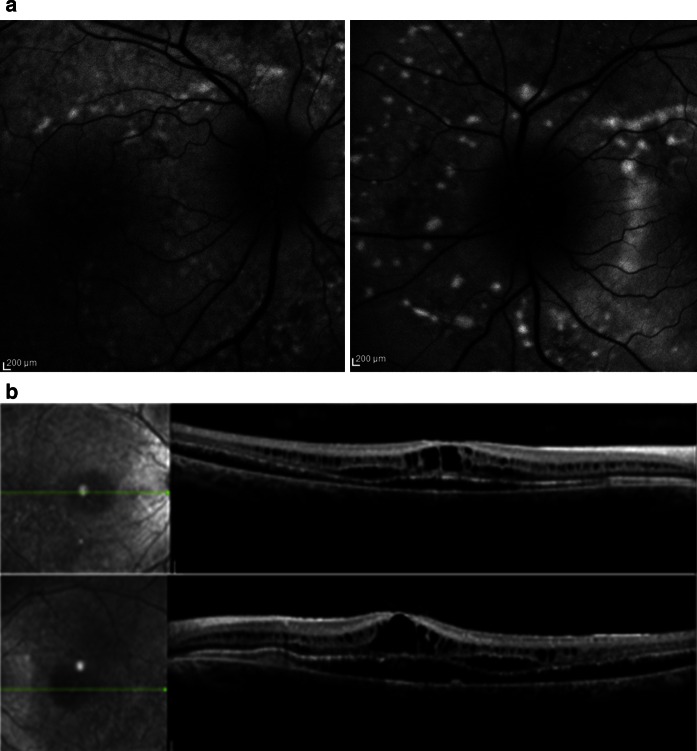
**a** *Right* and *left* autofluorescent images demonstrating widespread autofluorescent lesions typical for ARB. **b** *Right* and *left* spectral domain OCT images demonstrating extensive retinoschisis, intraretinal and subretinal fluid

He was referred to us for a second opinion 12 years later. Best-corrected visual acuity (BCVA) was 6/36 right and 6/60 left. He had minimal refractive error of 0.5 dioptre spherical equivalent hyperopia in each eye. Intraocular pressure (IOP) was 13 mmHg right and 16 mmHg left. Fundoscopy revealed normal optic discs but a grossly abnormal retina bilaterally, with widespread subretinal yellow lesions which autofluoresced (Fig. 2a) and schitic changes at the foveae (Fig. 2b). Electrophysiology had deteriorated significantly and now demonstrated delayed and reduced rod responses, further delayed photopic and flicker ERG and reduced P50 in the pattern ERG and a grossly abnormal multifocal ERG. The EOG remained grossly abnormal (Fig. 1b). Testing for possible infective and inflammatory conditions was negative.

**Fig. 3:**
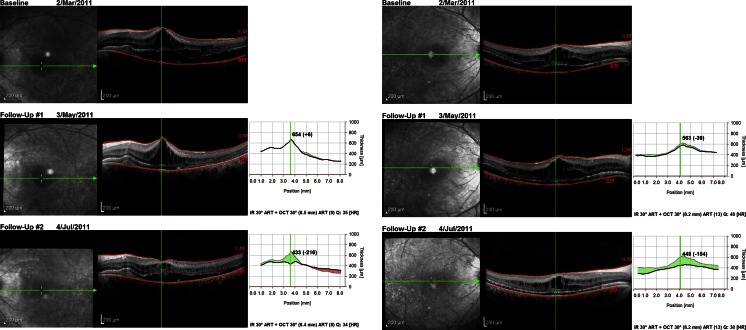
*Right* and *left* spectral domain OCT images demonstrating redistribution of intraretinal and subretinal fluid after 4 months of oral acetazolamide

A provisional diagnosis of ARB was made, and a trial of oral acetazolamide initiated in the light of reports of improvement in schitic changes in retinal dystrophies with carbonic anhydrase inhibitors [1]. Anatomical improvement in the central retinal thickness was noted whilst on treatment over a 6-month period. BCVA improved to 6/18 right eye, with no change in that for the left (Fig. 3).

The day after one of his follow-up visits during which dilated fundoscopy and retinal imaging was performed, and the dose of his acetazolamide was halved to 250 mg twice daily due to mild systemic side effects, he returned for unscheduled review, reporting left eye discomfort and increasing floaters. Visual acuity was unchanged. However, left IOP was markedly raised at 38 mmHg, compared with 4 mmHg for his right eye. A narrow drainage angle was noted bilaterally, but it was significantly narrower in the left eye, with a shallow anterior chamber and ‘volcano sign’. Subacute angle-closure glaucoma was diagnosed and effectively treated initially with topical anti-hypertensives and peripheral iridotomies. However, left-sided IOP continued to rise over a few days to 60 mmHg. B-scan ultrasound excluded choroidal effusion and confirmed plateau iris. His axial length measured 21.64 mm right and 22.06 mm left, and his anterior chamber depth was 2.48 mm right and 2.47 mm left. His optic disc was not cupped, with a cup-to-disc ratio of less than 0.4 bilaterally. Phacoemulsification lens extraction was eventually required to lower the IOP, and a 24.0 dioptre posterior chamber lens (A-constant 119.0) was inserted uneventfully. IOP was controlled at 18 mmHg thereafter but required continued use of topical prostaglandin and beta-blocker.

Bi-directional DNA sequencing of coding and flanking intronic regions of *BEST1* revealed that the patient was homozygous for a novel splice variant, c.636+1 G>A. This substitution was predicted to be pathogenic by abolishing the conserved donor splice site (Mutation Taster; Human Splicing Finder v2.4.1; NN SPLICE) [2], with potential usage of a cryptic donor splice site present 294 bp downstream in intron 5 (0.88 NN SPLICE). Cascade family testing identified this patient’s non-consanguineous parents and sister as carriers. They were asymptomatic and did not show signs of disease, with completely normal vision, fundi, anterior segments and electrophysiology.

## Discussion

The *BEST1* gene, formally known as *VMD2*, encodes bestrophin-1, previously postulated to act as a Ca^2+^-activated chloride channel [3], a regulator of voltage-gated Ca^2+^ channels [4], or a HCO_3_^−^ channel [5] in the basolateral membrane of the RPE [6]. It was recently shown to localise in the endoplasmic reticulum membrane, however [7]. Bestrophin-1 dysfunction has been associated with defective regulation of subretinal fluid reabsorption and aberrant phagocytosis of the photoreceptor discs [8]. Over 250 disease-causing mutations have been identified in the *BEST1* gene to date associated with a broad range of phenotypes, including BVMD, adult vitelliform macular dystrophy, autosomal dominant vitreoretinochoroidopathy (ADVIRC), the MRCS (microcornea, rod-cone dystrophy, cataract, posterior staphyloma) syndrome, retinitis pigmentosa and ARB [9–14].

ARB is thought to result from biallelic functionally null mutations of the gene, whilst most dominantly inherited missense mutations have been found to produce dominant negative effects and so do not compromise protein synthesis [10, 12, 13]. In vitro studies using HEK293 cells showed that co-transfection of the two mutations observed in the compound heterozygous state in ARB abolished chloride conductance in contrast to co-transfection of a single mutant with wild-type bestrophin-1 which led to significantly smaller chloride currents compared to wild-type bestrophin-1 [9, 15]. This suggests that the autosomal recessive phenotype only manifests when bestrophin-1 activity falls below a functional threshold. Davidson et al. [15] also found that different ARB-associated mutants lead to the same disease phenotype but through different effects on cellular processing mechanisms. This finding has implications for potential gene replacement therapies as the authors showed that missense mutations associated with autosomal recessive diseases may have a pathogenic outcome beyond simple loss of function.

Whilst BVMD is characterised by vitelliform lesions that typically occur at the macula as a result of abnormal deposition of lipofuscin in the retinal pigment epithelium (RPE), ARB is associated with subretinal deposits occurring predominantly outside the macula, mainly at the posterior pole and along the vascular arcades [9, 16–19]. These are often small and fleck-like or punctate in shape, white or yellow in colour, and hyperfluoresce on fundus autofluorescence imaging. Optical coherence tomography (OCT) shows subretinal and intraretinal fluid accumulation, often at the maculae [9, 18–21]. Using higher resolution Fourier-domain OCT, Gerth et al. [17] demonstrated RPE deposits and significant photoreceptor changes but preserved inner retinal layers in an 11-year-old boy with ARB, including thickened, elongated photoreceptor outer segments and detachment from the RPE.

Unlike BVMD, ARB is associated with diminished rod- and cone-driven ERG responses, though it shares the presence of a severely reduced or absent EOG light rise with BVMD as well as ADVIRC [9, 10, 16–24]. The expression of *BEST1* is higher in the peripheral RPE than at the macula [25]. This may explain the more widespread and progressive photoreceptor dysfunction, as well as the predominantly peripheral location of retinal lesions observed in patients with ARB. No histopathological data are available due to the novel description of the ARB phenotype.

Since ARB was first recognised in 2008 by Burgess et al. [9], at least 35 novel compound heterozygous and homozygous mutations have been reported to cause the disorder [9, 16–19, 21, 26–31]. Incomplete penetrance and variability in expression associated with some mutations in *BEST1*, together with the fact that some phenotypic distinctions may not develop until later years, make it difficult to distinguish those resulting in dominant inheritance from those resulting in recessive inheritance.

A family reported by Schatz et al. in 2006 [32] with compound heterozygous *BEST1* mutations is thought by some to have represented ARB rather than ‘atypical’ BVMD [9]. However, the heterozygous carriers also had ERG and EOG abnormalities, suggesting a diagnosis of BVMD with reduced penetrance in those individuals. Sharon et al. [30] recently reported a novel *BEST1* mutation in a Danish family in which the proband also had a previously reported mutation of the other allele and a phenotype suggestive of ARB. However, his mother and sister who were heterozygous for the novel mutation additionally had reduced EOG light rise which would not be expected with an autosomal recessive inheritance pattern. Pineiro-Gallego et al. [28] similarly reported on a case with a homozygous mutation causing what they considered to be ARB even though heterozygotes in the family also had abnormal EOG findings. One of the two cases of ARB reported by Pomares et al. [29] had fundus findings suggestive of ARB, but the patient had a normal EOG and no family history given he was adopted and so his novel homozygous mutation cannot be confirmed to cause ARB.

Bitner et al. [33] were of the impression that BVMD can be inherited as an autosomal recessive disease and distinguished this from ARB where the patients were homozygous for a novel mutation, had fundus and electrophysiology findings in keeping with a diagnosis of BVMD and their unaffected parents each carried one copy of the same mutation. As shown by Cascavilla et al. [26] however, it may not be until later years that the ERG becomes abnormal in cases of ARB.

In addition to small eyes, reduced axial length and hyperopia, it is increasingly recognised that bestrophinopathies are also strongly associated with anterior segment abnormalities and a high incidence of narrow-angle glaucoma [9, 10, 13, 14, 20, 22–24, 34]. The causes behind these associations are still to be elucidated, but there is evidence to support the hypothesis that *BEST1* is involved through bestrophin-1 expression in the RPE in the development of ocular structures beyond the retina [10]. Wittstrom et al. [22] reported that two of four patients with BVMD from one pedigree exhibited shallow anterior chambers, and all four patients had hyperopia as well as reduced axial lengths. Micropthalmos (axial length ≤20 mm) was found in two cases, both of whom had narrow angles and one of whom developed acute closed-angle glaucoma at the age of 12 years. Low et al. [34] found that sibling carriers of probands with BVMD can also have narrow anterior chamber angles, short axial lengths and a similar risk of angle-closure glaucoma.

Angle-closure glaucoma has been shown to affect approximately 50 % of those with ARB. Burgess et al. [9] found that all seven cases of ARB examined were hyperopic, and three required surgery for angle-closure glaucoma. Davidson et al. [20] described two unrelated cases of ARB, both of whom had angle-closure glaucoma contributing to their visual loss. All ten patients with ARB examined by Boon et al. [18] were hyperopic, and five had shallow anterior chambers and narrow angles for which they underwent prophylactic laser peripheral iridotomies. One of these patients with additional short axial lengths underwent phacoemulsification and intraocular lens implantation in an effort to deepen the anterior chamber and its angles for both eyes. Unlike the case we have presented, this was not enough to control her IOPs and she went on to have an iris base laser iridoplasty followed by topical anti-glaucomatous therapy. The authors postulated that cataract extraction may not sufficiently open the anterior chamber angles as some patients with ARB may have a dysgenesis of the anterior segment that additionally affects the trabecular meshwork.

Although plateau iris has not been described previously in ARB, a study by Etter et al. [35] suggests that it has a heritable component, with five of the ten patients studied with plateau iris syndrome having at least one affected first-degree family member. The authors likened this to an autosomal dominant pattern of inheritance with incomplete penetrance.

In conclusion, we report a novel *BEST1* splice variant, c.636+1 G>A, identified homozygously in a proband clinically diagnosed with ARB in association with plateau iris and narrow-angle glaucoma. A heterozygous G>C change in this position has been reported before, together with known pathogenic variant c.422 G>A (R141H) for a patient clinically diagnosed with BVMD [36]. The splice variant reported in the present study is likely to abolish the conserved 3′ splice site of exon 5. Family members carrying one copy of the mutation had no symptoms or signs of disease, supporting a recessive inheritance pattern; however, functional studies are required to confirm pathogenicity of the variant. We strongly recommend routine screening for narrow angles and glaucoma in ARB and BVMD given their frequent association, as well as close monitoring of IOP even after peripheral iridotomy and/or phacoemulsification have been performed.
